# Making the most of X‐ray reject analysis in the digital age

**DOI:** 10.1002/jmrs.680

**Published:** 2023-04-16

**Authors:** Michael J. Neep

**Affiliations:** ^1^ Department of Medical Imaging Logan Hospital Meadowbrook Queensland Australia; ^2^ School of Clinical Sciences Queensland University of Technology Brisbane Queensland Australia

## Abstract

This editorial discusses reject analysis in the digital age. It provides some recommendations that need to be considered when designing and implementing a contemporary reject analysis process.

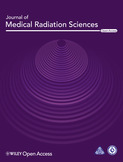

Some of you may still remember how to reject analysis was conducted back in the film‐screen days of general radiography. I can clearly picture sitting with my senior radiographer, circa early 2000s, while they reviewed on a lightbox, a pile of discarded X‐ray film. Prompted by my senior radiographer, we delved into a discussion on why I thought the X‐rays were rejected and how improvements could be made if I was to repeat the imaging. A paper notebook marked with a hand‐drawn ledger sat opposite the lightbox, documenting these discussions, and collating the anatomical region with the rationale for rejection. The reject count was then divided by the total number of films used during the period to determine the reject rate. Fast forward to the digital age of general radiography, the collation of quantitative reject data is virtually automatic on several manufacturers' X‐ray systems. Reject data can easily be exported in spreadsheet format providing additional information such as overall re‐exposure ratio and number of rejects per anatomical region. Despite this, frequently missing from contemporary reject analysis is the qualitative review of a rejected image. Examination of the rejected image is recommended as this can provide relevant information regarding problems with radiographic technique which exported data or overall reject ratios may not demonstrate. This component of the reject analysis process is frequently not performed in the digital age, primarily due to the challenges in accessing rejected images. In my opinion, this is where the most benefit is generated in the reject analysis process. The overarching purpose of undertaking reject analysis is to highlight areas of radiographic practice that can be improved upon, aiming to reduce unnecessary radiation dose to the patient.^1^ Undertaking reject analysis is not only beneficial to radiographers, but it is also valuable to students who are particularly focusing on honing their radiographic skills. By examining the underlying causes for image rejection and the individual examination types, it is possible to target specific projections that require optimisation with focused departmental and university education.

It was once thought that reject analysis was no longer necessary in the digital age. This was due to studies from the film‐screen era documenting that a major contribution to rejected images was attributed to incorrect exposures. Due to the dynamic range of digital radiography in comparison to film‐screen technology, it was proposed that reject rates could be eliminated. While the transition to digital radiography reduced the number of exposure‐related rejected images, rejected images did not disappear. To a large extent, the exposure‐related issues of film‐screen technology have now been replaced with patient positioning errors. These findings are supported in this edition of *Journal of Medical Radiation Sciences* (JMRS), where Bantas et al.^2^ conducted a multi‐site study of digital radiography reject rates and reject rationales in 2021 in two New Zealand radiology departments. They reported that patient positioning was found to be the most common rationale for image rejection and calculated overall reject rates of 7.86% and 5.91% for each site. Despite these reject rates being considerably lower than reported rates for film‐screen departments which have been documented to be between 10% and 15%,^3^, ^4^ these findings further support the need for reject analysis in the digital age. Additionally, Bantas et al.^2^ highlighted that it would be beneficial to include a review of radiographic positioning technique within the reject analysis process.

To make the most of undertaking reject analysis in digital radiography, I recommend considering the following factors when designing your reject analysis process. Some of which include, sole reliance upon overall departmental reject analysis rates, ease of access to rejected images, and image quality standards, in addition to, appropriate reject criteria and rationales.

Reliance upon the overall reject rate of a radiology department may camouflage problems in individual examinations whereby aggregated analysis can hide root causes. By limiting reject analysis to maintaining a target rate, potential opportunities for quality improvement are lost. In addition to calculating an overall reject rate which acts as a global quality indicator for a radiology department, it is pertinent to analyse the type of radiographic examination, examine the rejected rationale and review the rejected image. Thankfully, the first two of these three factors can be easily exported in spreadsheet format for sorting and analysis purposes.

Reject rates in isolation do not provide actionable information. To ascertain a clear understanding of why an image was rejected, one really needs to review the rejected image along with the rejected data. Unfortunately, not all vendors reject analysis programs provide easy access or access at all to the rejected images and corresponding data. This highlights a significant gap in current technology. Vendors need to further develop their reject analysis software so that easy access to rejected images and reject data is possible.

Given that X‐ray image quality is determined visually, it can be deemed inherently subjective. When reviewing rejected images, some choices to reject an image are obvious, such as missing significant anatomy. However, some choices are less obvious and require more careful judgement, for example, mild patient rotation. The subjective nature of the visual image quality assessment can lead to inconsistencies among radiographers in deciding whether to reject or repeat an image. In addition to the radiographer's assessment of image quality, radiologists tend to differ with variable tolerances of image quality acceptability. In this edition of JMRS, Decoster et al.^5^ conducted a multisite study involving three countries that sought to explore the difference between radiographers and radiologists based on their judgement on the quality of a radiograph. They found that radiographers were more critical in their assessment of radiographs compared to radiologists. This finding correlates with the findings of multiple previous studies.^6^, ^7^ This raises the question as to whether images are being prematurely rejected. Furthermore, I believe that the reject rates in digital radiography can also be attributed to the ease of repeating an X‐ray. This practice can be thought of as ‘Reject Creep’ in a similar way to the phenomena of exposure and dose creep,^8^ which has been widely reported since the introduction of computed radiography. In comparison with film‐screen technology which involved manual processing of a cassette between exposure and viewing the resultant image, digital radiography has no manual intervention between exposure and acquisition of the resultant image. This ease of acquiring an image and thus repeating an exposure may change the radiographer's decision on whether it is necessary to improve image quality or not. To combat the variance in image quality acceptability among radiographers, I recommend developing a document that clearly outlines acceptable image quality and tolerance for all anatomical regions. This is best developed locally and collaboratively with radiologists and radiographers so that a mutual standard can be achieved. This would aim to mitigate radiographers prematurely rejecting an X‐ray that a radiologist may consider acceptable. Finally, I have found it valuable to include a sample of accepted images for an overall quality review.

The final factor to consider when setting up your reject analysis process is to ensure consistent and accurate reject rationales are available for radiographers to select following the decision to reject an image. Vendor reject analysis programs vary in what reject rationales are available to select. Additionally, some vendor programs do not support a customizable list of reject rationales. This can lead to reject rationales that may not accurately reflect the rationale to reject. An example of this includes a reject rationale of ‘incorrect detector selected’ which I have experienced as a common cause of a rejected image in digital radiography. Furthermore, if multiple rationales to reject an image occur, for example, missing anatomy and patient positioning, the radiographer may not be able to select multiple rationales to reject. Therefore, resulting in inconsistent recording of reject data, which may hinder opportunities for quality improvement. I recommend consulting with the vendor to ensure the list of possible rejected rationales is consistent across all systems and is accurate to record all the possible rationales to reject an image.

In summary, reject rate analysis in isolation may not easily translate into measurable quality improvements. I believe that by a thorough examination of rejected images, standardising image quality assessment criteria, and ensuring reject rationales are consistent and accurate as part of the reject analysis process, we can provide the basis from which to develop targeted training programs. This in turn is likely to improve the overall image quality and dose reduction in the digital age of radiography.

## Conflict of interest

The author declares that they have no competing interests.

## Data Availability

Data sharing not applicable–no new data generated.
